# A Stress Reduction Program Adapted for the Work Environment: A Randomized Controlled Trial With a Follow-Up

**DOI:** 10.3389/fpsyg.2018.00668

**Published:** 2018-05-09

**Authors:** Shirley S. Lacerda, Stephen W. Little, Elisa H. Kozasa

**Affiliations:** ^1^Hospital Israelita Albert Einstein, São Paulo, Brazil; ^2^Centro de Vivência em Atenção Plena, São Paulo, Brazil; ^3^Department of Health, Universidade Nove de Julho, São Paulo, Brazil

**Keywords:** stress, mindfulness, depression, anxiety, attention, work, mental health

## Abstract

**Objective:** The aim of this study was to evaluate an *in situ* stress reduction program, named PROGRESS, developed to meet the specific needs of workers in a business context and to research its impact upon non-severe psychiatric symptoms, stress, anxiety, depression, processing speed/attention and mindfulness.

**Methods:** Participants with stress complaints were randomized into two groups: the main intervention group: group 1-G1, (*n* = 22); and the control group: group 2-G2, (*n* = 22). The protocol was divided into three distinct phases for the purpose of the study. Both groups were evaluated at time 1 (T1), before the first 8-week intervention, which only G1 received. The second evaluation was made on both groups at time 2 (T2), immediately after this first program; in order to test the program’s replicability and investigate possible follow-up effects, an identical second 8-week program was offered to G2 during time 3 (T3), while G1 was simply instructed to maintain the practice they had learned without further instruction between T2 and T3. A Confirmatory factor analysis (CFA) was conducted to investigate the construct validity of PROGRESS.

**Results:** Repeated measures MANOVA test, comparing G1 and G2, showed the effect of the intervention from T1 to T2 (*p* = 0.021) and from T2 to T3 (*p* = 0.031). Univariate analysis showed that participants from G1 improved levels of non-severe psychiatric symptoms, anxiety, depression, stress, processing speed/attention and mindfulness when compared with G2, from T1 to T2 (*p* < 0.05). After the participants in G2 received the intervention (T2 to T3), this group also showed improvement in the same variables (*p* < 0.05). At the end of their follow-up period (T2-T3) – during which they received no further support or instruction – G1 maintained the improvements gained during T1-T2. The two main components were stress (stress in the last 24-h, in the last week and last month) and mental health (non-severe psychiatric symptoms, depression, anxiety and mindfulness).

**Conclusion:** PROGRESS, an *in situ* mindfulness program adapted to fit within the reality of business time constraints, was effective at replicating in more than one group the reduction of stress, depression, anxiety, non-severe psychiatric symptoms, processing speed and also the improvement of attention skills, showing sustained improvement even after 8-weeks follow-up. Clinicaltrails.gov identifier: NCT02660307. https://clinicaltrials.gov/ct2/show/NCT02660307?term=Progress&rank=6

## Introduction

### Stress at the Workplace

The American Psychological Association survey on stress in the United States conducted in 2015 (American Psychological Associaton, 2016), revealed that the relationship between money and work were the top two sources of significant stress. Participants also reported stress as a source of negative impact on their mental and physical health, and a considerable proportion of them did not feel they were doing enough to manage it.

The National Institute of Occupational Safety and Health in the United States reported that 25% of American employees mentioned that their jobs are the main stressor in their lives and 40% considered that their jobs are very or extremely stressful (Centers for Disease Control and Prevention, 2017). It is well known that stress is associated with health problems, such as mental and cardiovascular disorders, and a cause of absenteeism and reduction in productivity in companies (Bhui et al., 2012; Henderson K. M. et al., 2013).

Chronic stress at work may lead to burnout, with detrimental consequences to workers’ well-being and health. In a systematic review burnout was shown to be a significant predictor of physical consequences such as coronary heart disease, cardiovascular disorders, musculoskeletal pain and prolonged fatigue. As for the professional impact, the review related burnout to dissatisfaction, absenteeism and presenteeism. Among psychological symptoms, the study found depression, insomnia and psychological ill-health. Both preventative interventions and early identification of burnout in the work environment may alleviate the individual and social impacts of this condition (Salvagioni et al., 2017).

Depression and anxiety are common comorbidities associated with stress. In their review, Liu and Alloy (2010) discussed the relation between stress and depression. Their review suggests that earlier research demonstrated stressful events often triggering symptoms of depression and anxiety, whereas more recent research revealed that depression and anxiety can increase future exposure to stress. This suggests that stressors and internalizing responses influence each other reciprocally.

Work stress is not, unfortunately, related only to the work environment. Employees who fail to psychologically deal with stressful events in the workplace also experience difficulties in facing life outside working hours. According to Demsky et al. (2014), work stressors such as workplace aggression may be associated with higher levels of work-family conflict, a form of conflict in which pressures from work and family are mutually incompatible to some degree. It is associated with a number of important problematic outcomes, including negative employee attitudes and reduced performance, health and well-being. Work stress at the end of a workday is related to work-related rumination and low levels of restful sleep measured the morning after (Vahle-Hinz et al., 2014). On the other hand, stress reduction programs may improve well-being and lead to prosocial behaviors (Bhui et al., 2012; Davidson and McEwen, 2012).

### Mindfulness-Based Interventions

The increasing worldwide concern about the impact of stress-inducing lifestyles has a large part to play in the interest research has gained into mindfulness-based interventions. Currently, many of the most successful skills-based stress reduction programs are based on this principle of mindfulness. According to Kabat-Zinn (2003), mindfulness means paying attention in a particular way; on purpose, in the present moment, and non-judgmentally. It was Kabat-Zinn who, in the late 1970s, created the *Mindfulness-Based Stress Reduction* (MBSR) program which includes contemplative practices (such as meditation, gentle movement, and body awareness techniques), for the development of self-knowledge and resilience (Chiesa and Malinowski, 2011; Salmon et al., 2011). MBSR was originally developed for the management of chronic pain and stress-associated illness and it was primarily this program that paved the way for many recent innovations in contexts outside hospitals and health clinics. The original MBSR format comprises eight 2.5 – 3 h weekly sessions with a silent 7-h retreat (shortly after the sixth session). It is recommended that everyday during the program the participants do both formal silent meditative practices and also informal practices such as mindful walking or eating (Kabat-Zinn, 1990).

Since the very early days of secular mindfulness training it has been found that silent meditation practices can support the cultivation of mindfulness. The practitioner learns how to develop a broader inner perspective on their own passing thoughts and emotions, identifying them as simple mental events occurring in the present moment. Participants in these programs develop the ability to reduce their over-identification with these mental events even during stressful situations, instead of engaging in anxiety, preoccupation or in negative thinking which can lead into a cycle of stress reactivity (Kabat-Zinn, 1982; Teasdale et al., 1995). Some mindfulness-based programs were combined with Cognitive Therapy to improve these abilities. One of the most well-known of these if Mindfulness-based Cognitive Therapy which was developed to help patients diagnosed with major depression to learn to manage and prevent relapses (Teasdale et al., 2000).

The participants develop sustained attention to observe their thoughts and emotions without identifying themselves with them, observing the adaptive and non-adaptive content as soon they appear in the mind. With persistent practice in this way they begin to notice triggers from which these cognitive and emotional patterns are stimulated and to perceive their subsequent consequences. As a result, mindfulness helps to reduce the tendencies which charge these non-adaptive thoughts and emotions (Rapgay and Bystrisky, 2009), thereby reducing their negative effects, and increasing positive outcomes (Schroevers and Brandsma, 2010).

### Benefits of Mindfulness at the Workplace

Davidson et al. (2003) evaluated 48 biotech employees who received an in-situ MBSR protocol during their working hours, and the results suggested an increase in antibody response to a flu vaccine. Since 2003 comparatively few studies of this kind have been carried out to further the investigation of the possible benefits of mindfulness interventions within the business community (Hyland et al., 2015).

Mindfulness-based programs have been seen to be efficient in dealing with intrusive thoughts, rumination and stress (Mendelson et al., 2010) and in the prevention of relapse in depression (Segal et al., 2010). Mindfulness programs have been efficient in alleviating some of the secondary effects of chronic pain (Morone et al., 2008) and primary insomnia (Gross et al., 2011).

The investigation into whether mindfulness reduces emotional exhaustion and improves job satisfaction has also been carried out (Hülsheger et al., 2013). This research revealed that as a result of short mindfulness interventions (5 or 10-day trainings) – known as “low-dose mindfulness interventions” – mindfulness is positively related to job satisfaction, and negatively associated with emotional exhaustion.

A few years later the same team devised a self-training, randomized field protocol with a wait-list control group. They investigated psychological detachment, sleep quality, and sleep duration assessed in daily measurements over 10 workdays. The intervention had effects on sleep quality and sleep duration, however no effects were found for psychological detachment after work (Hülsheger et al., 2015).

A significant increase in the risk of injuries at work has been associated with fatigue, rush, distraction, emergency situations, teaching or being taught by someone, field, excess noise, complex procedures, anger, along with other factors (Valent et al., 2016). Most of these factors are related to the lack of attention to the work in progress which may be improved by mindfulness training. Jha et al. (2007) evaluated changes in attention using the Attention Network Test (ANT) devised to identify behavioral and neural indices of alerting, orienting, and conflict monitoring during a single task (Fan et al., 2002). The participants in the MBSR group significantly improved the aspect of attention known as orienting when compared to the control and retreat participants. The orienting system has been associated with areas of the parietal and frontal lobes (Posner, 1980). Tang et al. (2015) proposed in a review about brain regions and mindfulness, the involvement of anterior cingulate and striatum (attention control), prefrontal and limbic regions and striatum (emotion regulation), insula, medial prefrontal and posterior cingulate cortices and precuneus (self-awareness).

### Developing a Tailor-Made Mindfulness Program for the Workplace

Studies within the workplace focus on programs which range from 36 h of training – a mindfulness course aimed at relieving teachers of stress (Roeser et al., 2013) – to research of highly shortened interventions lasting between 5 or 10 days for employees (Hülsheger et al., 2013). Adapting these interventions to the needs of the workplace is the challenge of the present protocol.

This current study was developed around lengthy discussions in 2012 with the Brazilian National Confederation of Industries (CNI) and the Brazilian Institute of Social Services for Industry (SESI). These initial discussions led to the development of a new 9-h, 8-week mindfulness-based stress reduction in-situ training program, called PROGRESS. This format both respects the limiting time constraints of business, and also remains true to the nature of the mindfulness process, which defies any “quick fix” approach and requires time and patience to unfold.

This program was developed with three main aims in mind: providing psychological well-being (stress reduction and increased attention skills), developing emotional skills (such as empathy and interpersonal relating) and also capable of having classes short enough to fit into existing business time constraints and the program itself long enough to respect the necessary 2-month learning process coupled with the integration of the basic skills into daily life.

The most obvious adaptation was having the weekly meetings brief (the first and last sessions lasting 90 min, and the others only 60 min each). Next, the theoretical and practical examples chosen for the course material had to be related specifically to the workplace and stress reduction within that context, otherwise the focus would be quickly lost and time wasted. As many employees would find it difficult to have the necessary space or discipline at home to reproduce the meditations between one class and the next, the weekly meditation routine was organized within the workplace with a special allocated space for that activity and an audio system installed there. The meditation recordings were all reduced to half the original durations defined by MBSR to facilitate daily practice during a heavy work routine. The original MBSR format of including a 7-h practice retreat in week 6 was removed from the program as it was impractical, and replaced by a “Practice class” in Class 6. And interpersonal relationship management and empathy were emphasized especially in sessions 7 and 8, being considered as two key abilities in human relationships, and served to broaden the scope of the course, eschewing any tendency for it to fall into being a mere attention/concentration training program.

From these considerations, we hypothesized that a specially adapted 9-h, 8-week *in situ* mindfulness program based on mindfulness practice and basic emotional skills – offered in the workplace – could be effective in the reduction of stress, depression, anxiety and non-severe psychiatric symptoms, in the increase of mindfulness and processing speed and that these changes could be sustained within the normal daily work routine even without further instruction after 8 weeks follow-up.

The aim of this study was to evaluate an in-situ mindfulness program, named PROGRESS, offered to meet the specific needs of workers in a business context and its impact upon non-severe psychiatric symptoms, stress, anxiety, depression, and attention.

## Materials and Methods

### Design

This is a randomized controlled trial with a follow-up.

### Participants

**FIGURE 1:**
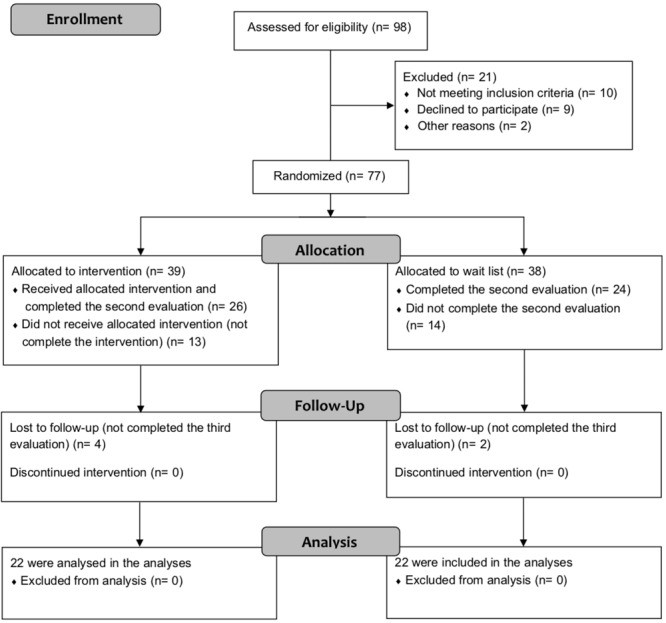
Flow diagram of recruitment of participants.

Seventy-seven participants (employees from two companies) agreed to participate in the study and were randomly divided in two groups (**Figure 1**). From the initial 77 participants, 44 were still participating at T2 and T3 in the study (22 in each group) and were evaluated at these points in the process: 20 (45.5%) were male and 24 (54.5%) were female, χ^2^(1) = 0.364, *p* = 0.546. There was also no significant difference in the proportion of male and female in each group, 9 (40.9%) males and 13 (59.1%) females in G1 and 11 (50%/50%) in each gender in G2, χ^2^(1) = 0.367, *p* = 0.545. Comparing the demographic scores and psychometric measures between groups, there were no significant differences between them (*p* > 0.05) (**Table 1**).

**Table 1:** Demographic characteristics in baseline of G1 and G2 the participants.

	G1 (*n* = 22)	G2 (*n* = 22)	Differences between groups at the baseline
	Average ± SE (Min – Max)	Average ± SE (Min – Max)	(*p*-value)^∗^
Age	35.68 ± 2.14 (19-53)	37.55 ± 2.06 (24 - 55)	0.534
Male (age)	35.11 ± 3.99 (19-53)	33.55 ± 2.51 (24-45)	0.735
Female (age)	36.08 ± 2.49 (21-51)	41.55 ± 2.89 (27-55)	0.163
SRQ-20	7.23 ± 0.83 (0-14)	6.64 ± 0.83 (1-19)	0.620
BDI	12.64 ± 1.50 (0-27)	11.45 ± 1.50 (2-30)	0.580
BAI	11.66 ± 1.78 (0-26)	12.27 ± 1.74 (1-32)	0.824
MAAS	57.28 ± 3.30 (29-84)	56.63 ± 3.22 (25-82)	0.928
Digit-symbol	64.84 ± 4.29 (38-91)	71.62 ± 4.68 (45-132)	0.095
ISSL (last 24 h)	3.54 ± 0.51 (1-9)	3.82 ± 0.51 (1-10)	0.709
ISSL (last week)	5.18 ± 0.62 (0-10)	5.31 ± 0.62 (1-13)	0.878
ISSL (last month)	5.59 ± 0.81 (0-13)	5.54 ± 0.81 (0-13)	0.968

*^∗^Student *t*-test; SE, Standard Error; n, Number of participants; SRQ-20, Self-Report Questionnaire; BDI, Beck Depression Inventory; BAI, Beck Anxiety Inventory; MAAS, Mindful Awareness Attention Scale; ISSL, Lipp Stress Symptoms Inventory.*

### Sample size

The sample size was calculated according to a confidence interval of 0.95, a sampling error of 0.05, and a power effect of 0.8. From this data, a sample size calculation was conducted and a minimum of 15 participants in each group was determined.

### Inclusion Criteria

We selected workers with stress complaints aging from 18 to 60 years. They had to be available to attend the program and also the before-and-after evaluations. As the questionnaires were self-administered, 8 years of education was necessary to assure that the participants were able to read and understand the questions of the scales and questionnaires.

### Exclusion Criteria

We excluded participants with a history of psychiatric or neurological disorders or who were under psychological or psychiatric treatment during the period of the study, or with a history of substance abuse, with the exception of tobacco.

### Procedure

**FIGURE 2:**
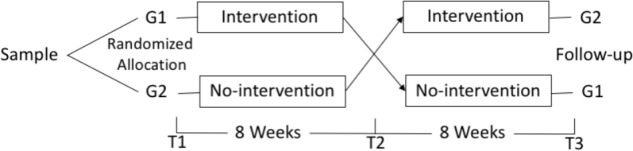
Study design.

The protocol was approved by the Universidade Nove de Julho Ethics Committee (CAAE 12585313.2.0000.5511) and registered at ClinicalTrails.gov (clinicaltrials.gov identifier NCT02660307). After signing an informed consent sheet, the participants with stress complaints were recruited in two companies identified by SESI (Serviço Social da Indústria – The Brazilian Institute of Social Services for Industry – a national organization) and the participants of each company were randomized according to a random number table into two groups: the PROGRESS group *N* = 22 (G1) and the control group *N* = 22 (G2) – G1 received the intervention between T1 and T2 while G2 received no orientation or intervention during this period. Both groups were evaluated before the intervention (time 1 – T1), after 8 weeks (after the period of the intervention for G1 – time 2 – T2); and at the end of another 8 weeks (when G2 received their own intervention while G1 were left to manage their practice on their own whilst continuing with the daily routine at the workplace – time 3 – T3). T3, therefore, served as a follow-up for G1 (see **Figure 2**).

### Questionnaires

- Self-Report Questionnaire-20 (SRQ-20): An inventory for the detection of psychiatric symptoms with 20 questions about mental health (Mari and Williams, 1985). The cut-off value was 7/8, with 86.33% sensitivity and 89.31% specificity (Goncalves et al., 2008). The score range is 0–20.
- Lipp Stress Symptoms Inventory (ISSL): To identify stress symptoms. This questionnaire measures stress “in the last 24 h,” “in the last week” and “in the last month” via 37 somatic symptoms and 19 psychological symptoms. The ISSL is divided in three independent scales and the cut-off number of symptoms for the first (stress in the last 24 h), second (stress in the last week) and third scales (the stress in the last month) were 7, 4 and 9, respectively. The ISSL internal consistency score measured by Cronbach’s alpha was 0.91 (Lipp, 2000).
- Beck Depression Inventory (BDI): The BDI has 21 items describing depression symptoms, each item in a scale from 0 to 3. The total score range is 0–63 and the cut-off value to discriminate mild to moderate symptoms of depression was 20 points, with 0.77 sensitivity and 0.95 specificity (Beck and Steer, 1984; Cunha, 2016).
- Beck Anxiety Inventory (BAI): The BAI has 21 items describing anxiety symptom, each item in a scale from 0 to 3 and the total score range is 0–63. The internal consistency was 0.91 and the test–retest reliability was 0.99 for a sample of the Brazilian population (Beck et al., 1988; Cunha, 2016).
- Digit-Symbol (DS WAIS- III): This test evaluates the processing speed and also visual and motor response by means of the association of numbers and symbols lasting 2 min. The test range 0–133 and the test–retest reliability was 0.84 for the Brazilian sample (Wechsler, 2004).
- Mindful Awareness Compassion Scale (MAAS – Mindful Awareness Attention Scale): In a scale from 1 to 6 the participant classifies how frequently or infrequently he/she becomes aware or mindful by contemplating the 15 described daily conditions provided (Brown and Ryan, 2003). The reliability scores for the Brazilian sample were 0.87 and 0.8 for internal consistency and test–retest, respectively (Barros et al., 2015).

### Intervention

#### PROGRESS – An *in Situ* Stress Reduction Program for Employees in Companies

This stress reduction program was structured around 8 classes – one class per week for 2 months. Whist the initial and final last 90 min – to allow for more dialog between the instructor and the participants – the remaining classes were designed to last 60 min. At the end of each session a printed handout and a cd, both with material relevant to that class, were distributed. Along with this material each participant also received a week-long diary to help them explore their practices and experiences. They were instructed to practice at least 5 times a week for up to half an hour a day at their workplace and were provided with a room at the company specifically dedicated for that purpose.

**1st Week- Mind-body interactions- being present:**

- Opening and introducing the program
- Short personal introduction from each participant
- Discussion about the expectations and basic guidance
- Practice: being present with the breath- 10 min

**2nd week- 5 ways to integrate presence into your life**

- Practice: Being present with the breath- 10 min
- Report-in: experiences from the week
- 5 ways to integrate presence into your life
- Practice: The 3-min pause

**3rd week- Perceiving body signals**

- Practice: The 3-min pause
- Practice: Being present with the breath- 10 min
- Perceiving body signals
- Practice: Body awareness

**4th week- What are my most common reactions?**

- Practice: The 3-min pause
- Practice: Body awareness
- What is my most common reaction?

**5th week- Dealing with Stress**

- A recap of the first four classes
- Practice: Being present with the breath – 5 min
- Report-in: experiences from the week
- New approaches to stress
- Evaluation: Half-way self-assessment
- Practice: Stop, Amplitude, Breath, Expand – SABE

**6th week- Practice class**

- Practice: Practice: The 3-min pause with SABE
- Practice: Being present with the breath – 5 min
- Practice: walking with presence

**7th week- Empathy**

- Presentation of a poem about repetition
- Reflection: tendencies that promote stress
- Empathy: why is it important?
- Explaining the empathy practice
- Practice: empathy

**8th week- Navigating during stress with wisdom**

- Sustaining everyday practice
- Practice: The 3-min pause - 3 min
- Practice: Being present with the breath – 5 min
- Practice: empathy
- Personal evaluation
- Training evaluation
- Report-in: my experience during the 8 weeks
- End of the training

**FIGURE 3:**
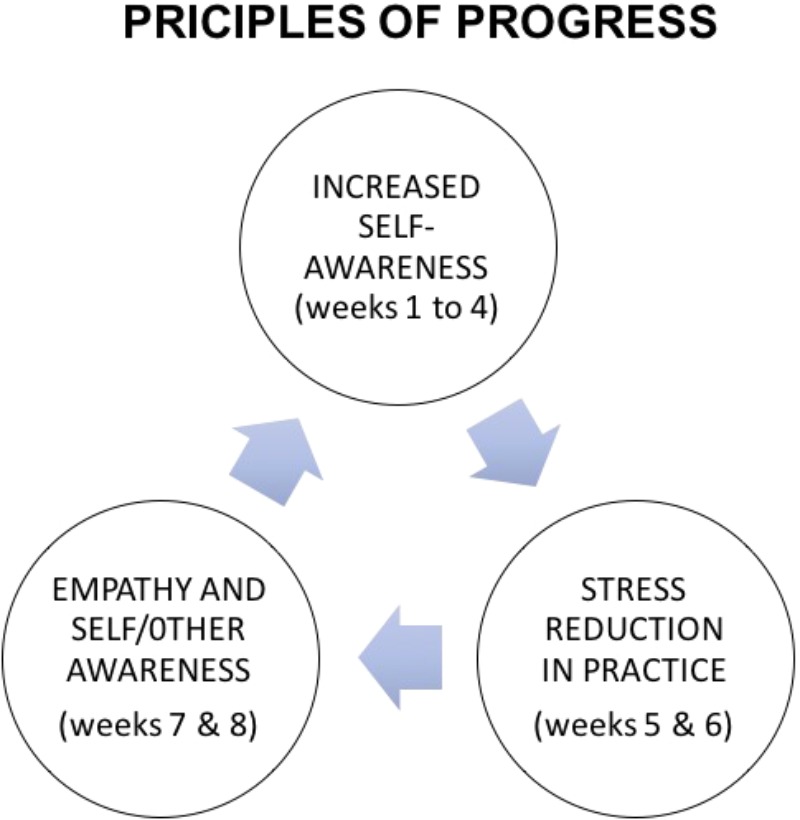
Principles of progress: the main elements of the program.

**Figure 3** is a schema of the principal phases of the Progress program. Weeks 1–4 are devoted to the development of self-awareness – awareness especially of palpable physical changes within the body during meditation and throughout the day. These first weeks are mainly dedicated to supporting the practitioner to recognize physiological and psychological reactions of stress, anxiety and depression in their mind and physical experience. In weeks 5 and 6 the participants learn, in increasingly specific ways, how to integrate into their daily lives the training principle of facing their stressful experience as it is happening, gradually reducing stress in practice. In weeks 7 and 8, they learn how to develop not only self-awareness but also self/other awareness which allows a more empathetic and constructive relationship with themselves and with other people. The schema is cyclic because we recommend participants to keep practicing after the end of the program.

### Statistical Analysis

The differences between the scores at baseline and those after the intervention were analyzed by a method of repeated measures. The differences between the groups was evaluated by the chi-square and Student’s *T*-test, while the comparisons between and within groups were analyzed by a MANOVA test for repeated measures.

A Confirmatory factor analysis (CFA) was used to investigate the construct validity of Progress. Several fit indices were selected to test which CFA model best represents the present dataset: root-mean-squared error of approximation (RMSEA), comparative fit index (CFI), chi-squared, and change in chi-square given the change in degrees of freedom between models.

Based upon the hypothetical underlying constructs for Progress, three models were developed to represent the best fit for the overall data. Model 1 was a one factor model used as a baseline comparison against the other models. Model 2 was a two-factor model with mental health and stress as latent factors. And the three latent factors of Model 3 were mental health, stress and attention.

The analysis was performed using the program IBM SPSS Statistics 22.0 (IBM) and IBM SPSS Amos Version 24.0 (IBM).

## Results

G1 and G2 were similar in all evaluated variables (see **Table 1**).

The repeated measures MANOVA test was conducted to test the effect of the intervention on variables over time. The results showed differences between G1 and G2 in non-severe psychiatric symptoms (SRQ), depression (BDI), anxiety (BAI), mindful attention (MAAS), processing speed (digit-symbol), stress “in the last 24 h” (ISSL last 24 h), “last week” (ISSL last week) and “last month” (ISSL last month) between T1 and T2, *F*(7) = 2.562, *p* = 0.021, $\eta_{p}^{2}$ = 0.304, OP = 0.867 and between T2 and T3, *F*(7) = 2.617, *p* = 0.031, $\eta_{p}^{2}$ = 0.456, OP = 0.819. As both groups received the intervention (G1 after T1 and G2 after T2), the results showed no difference between groups G1 and G2 in T1 and T3, *F*(7) = 0.352, *p* = 0.936, $\eta_{p}^{2}$ = 0.098, OP = 0.139. However, both groups improved from T1 to T3 in the different variables *F*(7) = 18.973, *p* < 0.001, $\eta_{p}^{2}$ = 0.854, OP = 1.000.

Univariate tests also indicate the intervention had an effect on variables over time. **Table 2** shows that, between T1 and T2, the effect of the intervention was significant when comparing G1 and G2 for all variables (*p* < 0.05) with a good effect size and acceptable observed power (ranged between 0.516 and 0.830). An intervention effect comparing groups G1 and G2 was also found between T2 and T3 for most of the variables measured, except for BAI, digit-symbol test and stress symptoms in the last 24 h (see **Table 3**). The intervention effect can also be observed in the comparison between T1 and T3 on time (**Table 4**) with a very good effect size and observed power, except for mindful attention (MAAS).

**Table 2:** Comparison between G1 and G2 in the baseline (T1) and after 8 weeks (T2), when G1 received the intervention.

	G1 (*n* = 22)		G2 2 (*n* = 22)		Time X Group Effect	Effect Size (Time × Group)	Observed Power (Time × Group)
	T1	T2^a^	T1	T2^b^			
			
	Average (*SE*)	Average (*SE*)	Average (*SE*)	Average (*SE*)	*p*		
SRQ-20	6.92 (0.78)	3.96 (0.72)	6.64 (0.67)	5.71 (6.48)	0.023	0.092	0.634
BDI	12.28 (1.60)	6.24 (1.20)	12.16 (1.43)	10.38 (1.07)	0.024	0.091	0.626
BAI	11.40 (1.69)	5.40 (1.56)	12.07 (1.52)	12.10 (1.40)	0.004	0.140	0.830
MAAS	58.72 (3.21)	65.08 (3.23)	57.58 (2.88)	57.25 (2.90)	0.029	0.085	0.595
Digit-symbol	62.92 (3.80)	73.96 (3.71)	68.26 (3.41)	69.19 (3.34)	0.047	0.071	0.519
ISSL (last 24 h)	3.36 (0.50)	1.76 (0.38)	3.23 (0.45)	2.87 (0.34)	0.047	0.071	0.516
ISSL (last week)	4.92 (0.59)	2.16 (0.52)	4.94 (0.53)	4.42 (0.47)	0.003	0.157	0.876
ISSL (last month)	5.40 (0.76)	2.24 (0.61)	5.00 (0.68)	4.16 (0.55)	0.010	0.116	0.743

*n, Number of participants; T1, baseline; T2, after 8 weeks; ^*a*^evaluation immediately after intervention; ^*b*^evaluation after 8 weeks without intervention; Size Effect, Partial Eta Squared (Less than 0.01 indicates a small effect size, 0.06 indicates a medium effect size and greater than 0.14 indicates a large effect size) (Cohen, 1973); SRQ-20, Self-Report Questionnaire; BDI, Beck Depression Inventory; BAI, Beck Anxiety Inventory; MAAS, Mindful Awareness Attention Scale; ISSL, Lipp Stress Symptoms Inventory.*

**Table 3:** Comparison between G1 and G2 (from T2 to T3), after G2 had also received the intervention.

	G1 (*n* = 22)		G2 2 (*n* = 22)		Time × Group Effect	Effect Size (Time × Group)	Observed Power (Time × Group)
	T2	T3^b^	T2	T3^a^			
			
	Average (*SE*)	Average (*SE*)	Average (*SE*)	Average (*SE*)	*p*		
SRQ-20	4.39 (0.86)	4.72 (0.71)	5.37 (0.92)	2.93 (0.75)	0.002	0.253	0.891
BDI	6.50 (1.53)	6.78 (1.09)	10.56 (1.62)	5.88 (1.57)	0.011	0.186	0.746
BAI	6.00 (1.87)	7.27 (1.47)	10.50 (1.94)	6.87 (1.56)	0.072	0.097	0.438
MAAS	62.61 (3.74)	60.27 (3.97)	55.43 (3.97)	62.63 (4.19)	0.009	0.196	0.773
Digit-symbol	75.00 (4.38)	83.78 (4.12)	73.19 (4.65)	83.19 (4.37)	0.826	0.002	0.055
ISSL (last 24 h)	1.83 (0.46)	1.72 (0.37)	3.19 (0.49)	2.19 (0.39)	0.231	0.044	0.220
ISSL (last week)	2.28 (0.61)	2.61 (0.44)	4.63 (0.65)	2.00 (0.46)	0.003	0.249	0.884
ISSL (last month)	2.16 (0.73)	2.33 (0.47)	4.43 (0.78)	2.00 (0.49)	0.003	0.250	0.886

*n, Number of participants; T2, after 8 weeks; T3, after 16 weeks; ^*a*^evaluation immediately after intervention; ^*b*^evaluation after 8 weeks without intervention; Size Effect, Partial Eta Squared (Less than 0.01 indicates a small effect size, 0.06 indicates a medium effect size and greater than 0.14 indicates a large effect size) (Cohen, 1973); SRQ-20, Self-Report Questionnaire; BDI, Beck Depression Inventory; BAI, Beck Anxiety Inventory; MAAS, Mindful Awareness Attention Scale; ISSL, Lipp Stress Symptoms Inventory.*

**Table 4:** Comparison between results of the participants in the baseline (T1) and after both groups had received the intervention (T3).

	G1 (*n* = 22)		G2 2 (*n* = 22)		Time Effect	Effect Size (Time × Group)	Observed Power (Time × Group)
	T1	T3^b^	T1	T3^a^			
			
	Average (*SE*)	Average (*SE*)	Average (*SE*)	Average (*SE*)	*p*		
SRQ-20	7.47 (0.90)	4.79 (0.68)	6.13 (0.98)	2.93 (0.74)	<0.001	0.395	0.995
BDI	13.21 (1.52)	6.68 (1.05)	10.56 (1.65)	5.87 (1.14)	<0.001	0.454	0.999
BAI	12.36 (1.82)	7.57 (1.43)	10.62 (1.99)	6.87 (1.56)	0.003	0.232	0.866
MAAS	55.42 (3.21)	59.16 (3.87)	59.13 (3.50)	62.63 (4.22)	0.092	0.083	0.391
Digit-symbol	64.84 (4.30)	83.00 (3.99)	71.62 (4.68)	83.18 (4.35)	<0.001	0.499	1.000
ISSL (last 24 h)	3.26 (0.52)	1.74 (0.35)	3.63 (0.57)	2.19 (0.39)	0.001	0.275	0.930
ISSL (last week)	5.00 (0.65)	2.58 (0.42)	5.19 (0.71)	2.00 (0.46)	<0.001	0.476	1.000
ISSL (last month)	5.53 (0.87)	2.26 (0.45)	5.50 (0.95)	2.00 (0.49)	<0.001	0.509	1.000

*n, Number of participants; T1, baseline; T3, after 16 weeks; ^*a*^evaluation immediately after intervention; ^*b*^evaluation after 8 weeks without intervention; Size Effect, Partial Eta Squared (Less than 0.01 indicates a small effect size, 0.06 indicates a medium effect size and greater than 0.14 indicates a large effect size) (Cohen, 1973); SRQ-20, Self-Report Questionnaire; BDI, Beck Depression Inventory; BAI, Beck Anxiety Inventory; MAAS, Mindful Awareness Attention Scale; ISSL, Lipp Stress Symptoms Inventory.*

**FIGURE 4:**
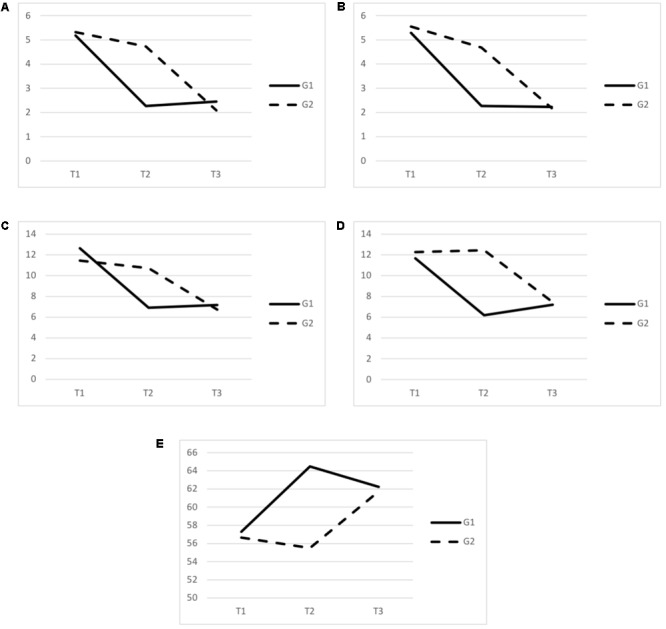
Comparison of the scores between G1 and G2 in the baseline (T1), after 8 weeks (T2) and after crossover (T3). G1 received the intervention between T1 and T2. G2 received the intervention between T2 and T3. **(A)** Stress scores in the last week; **(B)** stress scores in the last month; **(C)** depression scores; **(D)** anxiety scores; **(E)** mindfulness scores.

An overview of the results in G1 and G2 at T1, T2, and T3 can be seen in **Figure 4**.

**FIGURE 5:**
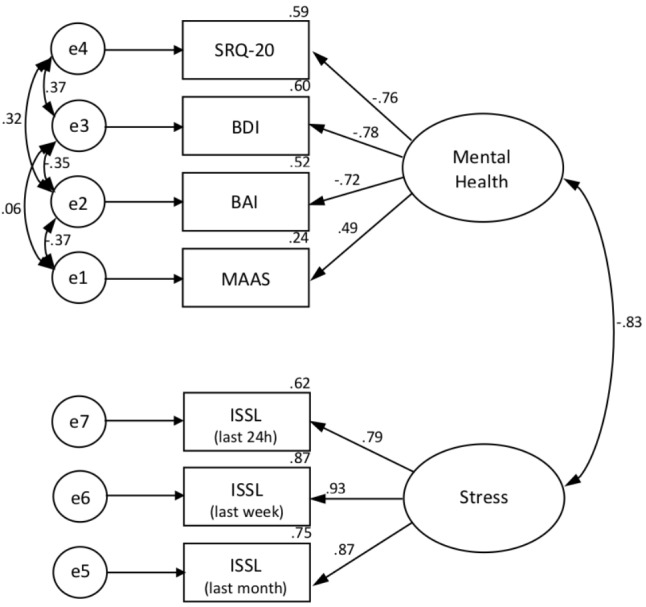
Confirmatory Factorial Analysis (CFA) for the 2 factors. SRQ-20, Self-Report Questionnaire; BDI, Beck Depression Inventory; BAI, Beck Anxiety Inventory; MAAS, Mindful Awareness Attention Scale; ISSL, Lipp Stress Symptoms Inventory; e, error.

A CFA was conducted to investigate the construct validity of Progress. The final sample size was 44 and there was no missing data. According to the fit indices, Model 2 was a significant improvement over Models 1 and 3. Model 2 had a lower RMSEA value (0.001), a higher CFI value (0.999), and a significant change in chi-square given the change in degrees of freedom when compared to Model 1 and Model 3 [χ^2^(6) = 4.692, *p* = 0.584] (see **Table 5**). No *post hoc* modifications were indicated in the analysis because of the good-fit indexes, and the residual analysis did not indicate any problems. From these results, Model 2 was selected as the best fit for the data (**Figure 5**).

**Table 5:** Fit indices for confirmatory factor models.

	RMSEA	90%CI	CFI	df	χ^2^	χ^2^/df	*p*
Model 1	0.052	0.001 – 0.129	0.991	11	8.418	0.765	0.675
Model 2	0.001	0.000 – 0.174	0.999	6	4.692	0.782	0.584
Model 3	0.362	0.307 – 0.419	0.756	36	1819.4	50.483	0.005

*RMSE, Root Squared Error of Approximation; CI, confidence interval; CFI, Comparative Fit Index; df, degree of freedom; χ^2^, Chi-square.*

## Discussion

We aimed to evaluate the effects of an 8-week in-situ mindfulness stress reduction program adapted for companies on non-severe psychiatric symptoms, stress, anxiety, depression and attention, while also investigation if the possible benefits would be sustained 8 weeks after the end of the program.

### The Efficacy of PROGRESS

The groups were similar at the baseline, therefore the statistical differences between the groups that appear after the intervention periods could be attributed to the training. The group which received the first intervention (G1) improved in all of the variables, and G2 – which did not receive it at that time – had no differences between the baseline and T1, suggesting that the intervention worked as we hypothesized, being effective in the reduction of stress, depression, anxiety and non-severe psychiatric symptoms and also increasing mindfulness and processing speed.

After G2 received the intervention it improved in the measured variables, except in BAI, digit-symbol and stress in the last 24 h. In the same period, participants in G1 were left to manage their practice on their own and they were able to sustain the improvements in the follow-up evaluation. This means that the learned skills were maintained without any further support or training in accordance with our hypothesis.

The improvements in both groups after their respective interventions in different time points were equivalent (there were significant differences from T1 to T3). It is important to notice that at the conclusion of the study (T3), after both groups had received the intervention, they had very similar outcomes.

PROGRESS participants showed reduction in non-severe psychiatric symptoms. Other interesting results were the increase in mindfulness and the increase in attention processing speed (digit-symbol results). This may considerably decrease the number of mistakes made during routine work and reduce injuries, which are a burden, especially in industry, and particularly in the case of one of the study settings. The low cost of stress reduction programs such as PROGRESS can open the possibility of investing in the human resources of the host company, and this may provide a return in productivity and reduced absenteeism (Henderson C. et al., 2013).

### Confirmatory Factor Analysis

As a complementary analysis, we conducted a CFA which showed that there are two main components responsible for the efficacy of PROGRESS: stress and mental health. Processing speed could not enter in the model because of the type of outcomes presented in this test.

The two main components were stress (stress in the last 24 h, in the last week and last month) and mental health (non-severe psychiatric symptoms, depression, anxiety, and mindfulness). Our hypothesis was that mindfulness would be a separate component associated with processing speed because both depend on attention, however mindfulness is more related to mental health than attention. On the other hand, as hypothesized, non-severe psychiatric symptoms, depression and anxiety were associated.

### Stress Reduction Programs

A number of successful stress reduction programs are based on the principle of mindfulness and involve self-knowledge and self-awareness (Chiesa and Malinowski, 2011; Salmon et al., 2011). PROGRESS is an adaptation of MBSR and, in it, mindfulness is taught to emphasize not only awareness of the self, but relational skills as well. The development of empathy may lead to a less stressful environment, and to a more cooperative predisposition within and between work teams and, of course, with the client. Most of the components of emotional intelligence (self-awareness, self-regulation, motivation, empathy, and social skill) (Goleman, 1998), are part of PROGRESS. These qualities are tending to be increasingly valued by both the employee and the organization. This ability to expand mindfulness into one’s everyday life has influenced how PROGRESS encourages the application of mindfulness practice within the work routine, and a key aspect of the program supports participants in coming up with their own ways of using the practice to manage the day-to-day work challenges they come up against. All of this is explored during the classes – especially in the second half of the course.

One common problem in organizations is that the individuals involved frequently avoid conflict due to the possible negative impact on the individual, group and the organization. Participants in a mindfulness program tended to decrease conflict avoidance and improve emotional acceptance compared with a control group (Skarlicki et al., 2015). Mindfulness practice arguably reduces over-identification with mental events and ruminations and it help to reduce negative thinking which frequently leads to the beginning of cycles of stress reactivity (Kabat-Zinn, 1982; Teasdale et al., 1995). Non-adaptive thoughts and emotions may be weakened by mindfulness practice (Rapgay and Bystrisky, 2009) and some evidence suggests a reduction in negative effects and an increase positive ones (Schroevers and Brandsma, 2010). A 3-week online self-training mindfulness intervention as a cognitive*–*emotional segmentation strategy to promote work*–*life balance compared to a waitlist control group showed promising results in promoting significantly less strain-based work*–*family conflict and significantly more psychological detachment and satisfaction with work*–*life balance (Michel et al., 2014).

Other mindfulness programs researched in companies report signs of more job satisfaction and reduced emotional exhaustion (Hülsheger et al., 2013), and better sleep quality (Hülsheger et al., 2015). These possible effects may explain the reduction of non-severe psychiatric symptoms, stress, depression and anxiety scores after the PROGRESS intervention.

According to Biggio and Cortese (2013), well-being in the workplace can be promoted not only “from above,” by means of actions from management level, but also “from below,” influencing individual traits and behaviors within the environment. This suggests that for a successful stress reduction program in companies it is important to have the participation of the different staff levels in the company, including leaders.

### Why PROGRESS Is Different From Other Programs?

PROGRESS was “tailor-made” for companies, and has deliberately adapted each class to a 1-h format (or an hour and a half in the case of the first and last classes). This shorter class time was specifically requested by The Brazilian Institute of Social Services for Industry – SESI, arising from the knowledge that a longer class time implies higher costs for companies and reducing employee adherence. A follow-up study, similar to a crossover design, was tested because it is important to verify if the participants were able to retain the benefits of the intervention, having only the written handouts and CDs as a support. Even without further instruction our follow-up has shown that the benefits were maintained. At the same time, we recommend companies to consider having regular practice classes as a sustaining support, maybe once a month, because they will probably have better results in the long term this way. Those who did not adhere to the program were interviewed revealing that the majority of these participants were unable to reorganize their schedule to the time-slot chosen by the company for the program to take place. In one company the allocated time-slot was scheduled early in the morning before the beginning of the working day. The other company defined the period before lunch as the best time for the program to take place. This points out the possible value of offering different training schedules within host companies. Taking these considerations into account we suggest that interventions based on mindfulness and the development of emotional abilities may be effective in reducing stress at the workplace.

There are few studies about mindfulness programs for companies. After interviewing employers and employees, PROGRESS was created in order to adapt a stress reduction program for companies. The adaptations we highlight are the brief and concise weekly class meetings, the additional support for short daily meditation practices within the company, and the emphasis on training interpersonal relationship management and empathy. The meetings are theoretical and practical with examples specifically related to the workplace and oriented to show how to learn to reduce stress.

### Limitations and Further Considerations

As a possible bias, one of the authors (SWL) developed this mindfulness program. PROGRESS was tested only in Brazilian industries and it may be a limitation of this study. Another limitation is the so-called Hawthorne effect: improvements can be related to participants knowledge about their allocation in the intervention or control groups (McCarney et al., 2007), however there are many discussions about the heterogeneity of study methods, contexts, and findings in Hawthorne effect research (McCambridge et al., 2014). Another limitation is the lack of a placebo group that could control the effect of the intervention expectation. Other interventions such as physical exercise or psychotherapy may reduce stress in the work environment, however we decided to focus our study on mindfulness which, by having a classroom format focused on training palpable skills over a short period of time, may be more easily implemented in a modern company setting. As a next step, we may suggest a larger sample in which it will be possible to compare the effects of PROGRESS on different categories of workers, such as leaders, administrative staff and factory production line employees. A placebo intervention could also be included and biological measures such as cortisol levels would also strengthen the evidence of the effects of PROGRESS. From the point of view of the company, it would also be important to measure productivity, absenteeism and presenteeism indicators, as well as job satisfaction.

Concluding, PROGRESS – when compared to a waiting-list – reduced non-severe psychiatric symptoms, stress, anxiety, depression and increased attention. Its efficacy can be attributed to its effects in two main components: stress and mental health. The improvements lasted at least for 8 weeks of follow-up.

## Author Contributions

SSL and EHK: Design, data collection, analysis, article writing, and revision. SWL: Intervention development, article writing, and revision.

## Conflict of Interest Statement

The second author is a mindfulness instructor. The other authors declare that the research was conducted in the absence of any commercial or financial relationships that could be construed as a potential conflict of interest.
